# IL-8 (CXCL8) Correlations with Psychoneuroimmunological Processes and Neuropsychiatric Conditions

**DOI:** 10.3390/jpm14050488

**Published:** 2024-05-03

**Authors:** Anton Shkundin, Angelos Halaris

**Affiliations:** Department of Psychiatry and Behavioral Neurosciences, Loyola University Chicago Stritch School of Medicine, Loyola University Medical Center, Maywood, IL 60153, USA; dr.shkundin@gmail.com

**Keywords:** IL-8, CXCL8, CXCR1, CXCR2, SNPs

## Abstract

Interleukin-8 (IL-8/CXCL8), an essential CXC chemokine, significantly influences psychoneuroimmunological processes and affects neurological and psychiatric health. It exerts a profound effect on immune cell activation and brain function, suggesting potential roles in both neuroprotection and neuroinflammation. IL-8 production is stimulated by several factors, including reactive oxygen species (ROS) known to promote inflammation and disease progression. Additionally, CXCL8 gene polymorphisms can alter IL-8 production, leading to potential differences in disease susceptibility, progression, and severity across populations. IL-8 levels vary among neuropsychiatric conditions, demonstrating sensitivity to psychosocial stressors and disease severity. IL-8 can be detected in blood circulation, cerebrospinal fluid (CSF), and urine, making it a promising candidate for a broad-spectrum biomarker. This review highlights the need for further research on the diverse effects of IL-8 and the associated implications for personalized medicine. A thorough understanding of its complex role could lead to the development of more effective and personalized treatment strategies for neuropsychiatric conditions.

## 1. Introduction

The intricate balance between the neuroendocrine and immune systems is critical for maintaining an organism’s homeostasis. During times of stress, the redistribution of immune cells across various immune compartments becomes crucial, ensuring the efficiency of cell-mediated immune responses [1]. The activation of the immune system in the periphery impacts the central nervous system (CNS), and disturbances in systemic immune functions can significantly contribute to the onset of neuropsychiatric conditions [2]. Dysregulation in neuroimmune interactions may lead to dysfunction in vital organs and result in the widespread impairment of neuromodulation and symptoms across multiple body systems [3]. Peripheral afferent neurons activated by the immune system, along with circulating cytokines and microbial products, stimulate neurons and glial cells in the hypothalamus and medulla, triggering sympathetic and humoral responses [3,4]. The gaining of insight into the comorbidities between psychiatric conditions and cardiovascular, cerebrovascular, and neurological disorders has been greatly enhanced through the perspective of psychoneuroimmunology, revealing the complex interconnections between the brain and the immune system [5,6].

Neuroinflammatory processes influence the health of the nervous system, exerting control over the development and viability of brain cells and their connections [7]. CNS immune responses can lead to synaptic dysfunction, neurotransmitter imbalances or deficiencies, neuronal loss, and the exacerbation of brain-related pathologies [5]. Neuroinflammation, implicated in a diverse array of CNS disorders, including autoimmune and degenerative pathologies, often manifests as a dysregulated chemokine system [7,8].

Chemokines, also referred to as chemotactic cytokines, comprise a diverse family of relatively small proteins, ranging from 8 to 12 kDa. These proteins are secreted and play a critical role in inducing chemotactic responses in immune cells, and participate in numerous inflammatory processes [9,10,11]. Their distinctive features lie in the presence of three to four conserved cysteine residues, which leads to their categorization into four families (CXC, CC, CX3C, and C) based on the arrangement of these N-terminal cysteine residues [12,13,14,15].

Chemokines play a key role in controlling the development and equilibrium of the immune system. They actively participate in all aspects of both protective and detrimental immune and inflammatory reactions [10]. These proteins can attract and activate a wide range of cells, including both immune and non-immune varieties [12,14]. Beyond their role in inducing chemotaxis, chemokines also exert control over cell proliferation, survival, and differentiation [16].

Chemokines and their receptors play a prominent role in facilitating communication between neurons and inflammatory cells, a crucial aspect of normal neuronal function [7]. These molecules and their receptors are naturally expressed in the brain, under physiological conditions. Their presence suggests their potential involvement in intercellular communication and the modulation of neuronal activity, in addition to their well-known immunological functions. Furthermore, chemokines are actively engaged in brain development and in the maintenance of brain homeostasis, influencing synaptic activities, as well as the processes of migration, differentiation, and proliferation in both glial and neuronal cells [8,14,17,18,19,20].

The recruitment of inflammatory cells is a well-recognized driver of the secondary damage cascades commonly observed in CNS injuries. Changes in the expression of chemokines drive the activation and infiltration of cells to the injury site. This post-traumatic infiltration of inflammatory cells has been linked to secondary tissue damage, cell death, and the demyelination of axons [21]. Furthermore, the potential of chemokines to induce neuronal death, either directly through the activation of neuronal chemokine receptors or indirectly by triggering microglial destroying mechanisms, underscores their significant role in CNS injury [7,8,13].

## 2. IL-8 and CXCR1/2 Receptors

Within the chemokine family, the CXC or α chemokines, are characterized by a single amino acid separating the first two cysteine residues, denoted as cysteine-X amino acid-cysteine (CXC). Among the CXC chemokines, there is a subgroup distinguished by the presence of a specific three-amino-acid motif near their N-terminal region, known as the glutamic acid-leucine-arginine (ELR) motif [12].

Interleukin-8 (IL-8), also recognized as neutrophil-activating peptide 1 (NAP1) and CXC chemokine ligand 8 (CXCL8) [22], is a proinflammatory CXC chemokine that plays a prominent role in inducing neutrophil chemotaxis, the release of intracellular granule contents, and the upregulation of cell surface adhesion molecules [23,24,25].

IL-8 belongs to the ELR+ CXC chemokine family, known for its diverse biological functions. It serves a critical role in guiding neutrophils and promoting angiogenesis [26]. IL-8 exists in two forms, a monomeric and a dimeric form, and this distinction can lead to different effects on its CXCR1/2 receptors, including desensitization and receptor internalization [26,27].

The action of IL-8 involves several cellular responses, including alterations in cytoskeletal structure, fluctuations in intracellular calcium concentrations, the activation of integrins, the release of granule proteins through exocytosis, and the initiation of a respiratory burst [28].

**Figure 1:**
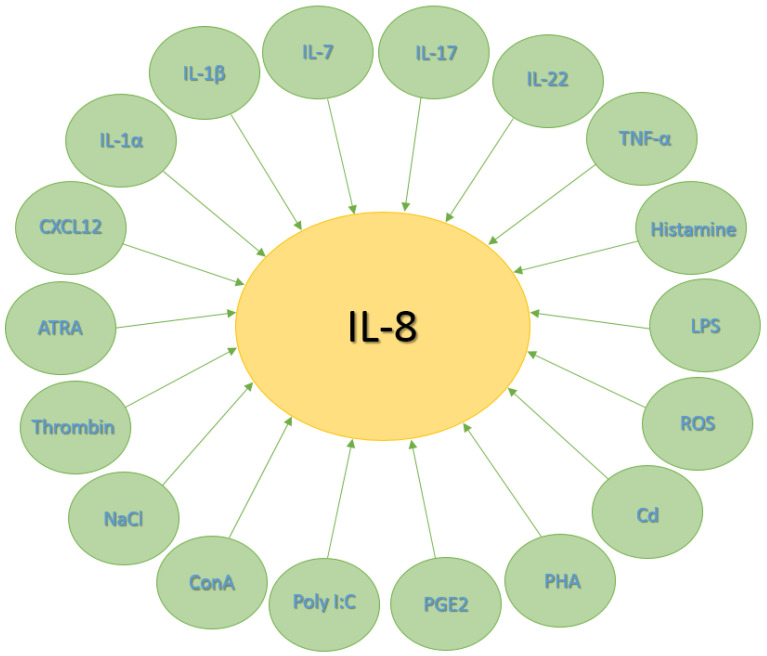
Schematic presentation of IL-8 inducers: IL-1α, IL-1β, IL-7, IL-17, IL-22, tumor necrosis factor-alpha (TNF-α), histamine, stromal cell-derived factor-1 (SDF-1, CXCL12), lipopolysaccharides (LPSs), reactive oxygen species (ROS), cadmium (Cd), phytohemagglutinin (PHA), prostaglandin E2, polyinosinic-polycytidylic acid (poly I:C), concanavalin A (ConA), NaCl, thrombin, and all-trans-retinoic acid (ATRA).

The production and release of IL-8 can be modulated by multiple factors that influence its expression and levels. For instance, IL-8 may be induced by several cytokines and substances, including IL-1α [29,30,31,32], IL-1β [30,33,34,35], IL-7 [35,36], IL-17 [35,37,38,39,40,41,42], IL-22 [39,40,41,43], tumor necrosis factor-alpha (TNF-α) [29,30,33,35,44,45,46], histamine [46,47,48,49,50], stromal cell-derived factor-1 (SDF-1, CXCL12) [51,52,53,54,55], lipopolysaccharides (LPSs) [33,35,44,56], reactive oxygen species (ROS) [57,58,59,60], cadmium (Cd) [61,62,63,64], phytohemagglutinin (PHA) [35,56], prostaglandin E2 (PGE2) [65,66,67,68], polyinosinic-polycytidylic acid (poly I:C) [35,69,70], concanavalin A (ConA) [35,71], NaCl [72,73,74,75], thrombin [49,76,77,78], all-trans-retinoic acid (ATRA) [79,80,81], and other cellular stressors (Figure 1).

**Figure 2:**
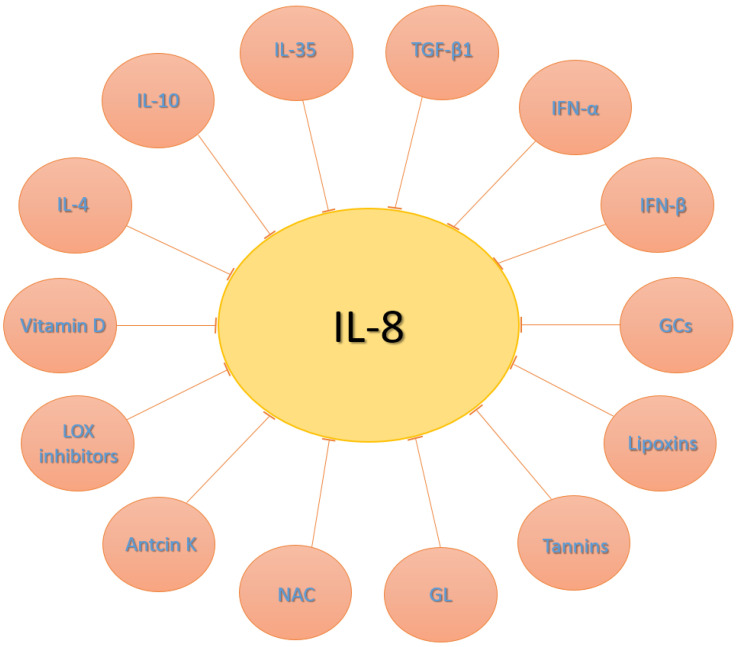
Schematic presentation of IL-8 reducers: IL-4, IL-10, IL-35, transforming growth factor-beta 1 (TGF-β1), interferon-alpha (IFN-α), interferon-beta (IFN-β), glucocorticoids (GCs), lipoxins, vitamin D, lipoxygenase (LOX) inhibitors, antcin K, tannins, glycyrrhizin (GL), and N-acetylcysteine (NAC).

Additionally, numerous other cytokines and compounds demonstrate the ability to reduce IL-8 levels, such as IL-4 [29,33,35,44,45,82], IL-10 [33,45,73,82,83], IL-35 [84,85,86], transforming growth factor-beta 1 (TGF-β1) [33,87,88], interferon-alpha (IFN-α) [82,89,90], interferon-beta (IFN-β) [82,89,90,91,92], glucocorticoids (GCs) [29,35,44,45,82,93], lipoxins [94,95,96], vitamin D [29,35,44,97,98,99], lipoxygenase (LOX) inhibitors [29,35,100,101], antcin K [102], tannins [103,104], glycyrrhizin (GL) [50,105,106,107] and N-acetylcysteine (NAC) [31,108,109,110] (Figure 2). It is important to note that these are not the only examples of IL-8 modulators, and their impact on IL-8 levels can vary depending on factors such as their concentration, duration of exposure, and the specific cellular context.

IL-8 displays remarkable resilience when exposed to temperature variations and proteolytic enzymes, and it maintains a relative resistance to acidic conditions, making IL-8 an exceptional candidate for deployment at sites experiencing inflammation, where it must endure harsh and adverse surroundings [111,112]. Unlike most inflammatory cytokines, which have a brief lifespan in vivo, IL-8 remains active for days or even weeks after its early production in the inflammatory response [111,112].

IL-8 exerts its effects by binding to specific G protein-coupled receptors known as CXCR1 and CXCR2 [25]. These receptors, belonging to the γ subfamily of G-protein coupled receptors with seven transmembrane domains, play an essential role in mediating IL-8’s effects [113]. Both CXCR1 and CXCR2 interact with IL-8, sharing significant amino acid sequence similarity and exhibiting a binding affinity for IL-8 [114,115,116].

However, these receptors show distinctions in their second extracellular loop, fourth transmembrane domain, C-terminal (intracellular), and N-terminal (extracellular) regions [114,115,116]. Moreover, their desensitization processes differ significantly, with CXCR2 internalizing more rapidly and at lower ligand concentrations compared to CXCR1 [114,116,117]. Additionally, CXCR2 undergoes recycling back to the cell surface at a significantly slower pace than CXCR1 [114,116,117].

Upon binding to CXCR1 and CXCR2, IL-8 induces calcium flows, chemotaxis, and degranulation. However, only CXCR1 is responsible for activating phospholipase D and the stimulation of the superoxide production through the NADPH oxidase enzyme, contributing to the respiratory burst and generation of reactive oxygen species (ROS) in neutrophils [27,114,118,119,120,121,122].

## 3. IL-8 and CNS

IL-8 plays a crucial role in the peripheral immune response, but it may also exert central effects and be involved in the regulation of neuroendocrine functions related to stress [123]. Glucocorticoids (GCs) have been shown to downregulate IL-8 mRNA expression [124] and decrease IL-8 serum levels [125]. Moreover, the CXCL8 gene features a glucocorticoid receptor binding core site situated at positions -330 to -325, making it susceptible to inhibition by glucocorticoids [123]. Interestingly, IL-8 mRNA expression was detected in the rat paraventricular nucleus of the hypothalamus (PVN), a key site for corticotropin-releasing hormone (CRH) synthesis, and the hippocampus, where negative feedback to CRH production is generated [17,123]. IL-8’s activation of the hypothalamic-pituitary-adrenal (HPA) axis increases cortisol production, which, in turn, helps protect the body from autoimmune disorders by suppressing proinflammatory cytokine production [126].

Additionally, IL-8 possesses the capability to influence glutamatergic synaptic transmission, impacting both presynaptic and postsynaptic processes. Increased IL-8 levels are implicated in notable alterations of synaptic transmission in the prefrontal cortex and may contribute to the development of persistent inflammatory pain [127].

Various cell types within the brain, including astrocytes, neurons, microglia, and endothelial cells, consistently express CXCL8 receptors [128]. The production of IL-8 has been noted upon the stimulation of microglia, resident brain tissue macrophages, and monocyte-derived macrophages (MDMs) [33]. These innate immune cells play crucial roles in the inflammatory response, the phagocytosis of cellular debris, and tissue repair following injury [129]. Furthermore, IL-8 is secreted by astrocytes, which are the most abundant cell types in the brain [33,130,131]. Astrocytes participate in numerous functions within the CNS, encompassing the regulation of glutamate, ion homeostasis (e.g., Ca^2+^, K^+^), and water balance, as well as the control of blood–brain barrier permeability, scar formation, tissue repair through angiogenesis and neurogenesis, and the modulation of synaptic activity [132,133]. IL-8 transcription in astrocytes is negatively regulated by β-catenin, and positively regulated by the interaction of T cell factors (TCFs), lymphoid enhancing factor (LEF), and activating transcription factor 2 (ATF2) [131].

IL-8 demonstrates strong trophic properties, guiding the movement and survival of neural stem cells and glial progenitor cells. Additionally, it facilitates glia-neuron communication by modulating neuronal excitability, triggering both excitatory and inhibitory activity [134,135]. The release of IL-8 by glial cells can activate CXCR1 and CXCR2 receptors on cholinergic septal neurons. This activation results in the immediate, direct, and reversible modulation of ion channels, leading to a reduction in Ca^2+^ currents through G-protein activation. Notably, cholinergic septal neurons, a neuronal type particularly vulnerable in patients with Alzheimer’s disease (AD), may be influenced by IL-8, potentially contributing to the cognitive deficits observed in these individuals [134]. Additionally, elevated levels of IL-8 in both plasma and cerebrospinal fluid (CSF) have been associated with increased CSF p-tau levels. Similarly, higher CSF IL-8 levels correlate with elevated CSF Aβ42 levels, while higher CSF sAβPPβ levels are linked to increased plasma IL-8 concentrations [136].

In the context of drug abuse, particularly Methamphetamine (METH) use, the importance of IL-8 and CXCR1 is exemplified. CXCR1 has been associated with the process of neuronal apoptosis induced by METH. The use of METH is linked to oxidative stress, the apoptosis of dopaminergic neurons, and neuroinflammation related to astrocytes [137]. Exposure to METH upregulates the expression of CXCR1 in neurons and amplifies the expression of IL-8 via the nuclear factor-kappa B (NF-κB) pathway in astrocytes. On the other hand, the suppression of CXCR1 expression using siRNA sequences notably mitigated METH-induced neuronal apoptosis and promoted the neuroprotective effect of astrocytes on neurons [137].

Furthermore, the neuroinflammation mediated by glial cells appears to exert an influence on cognitive aging. In healthy older individuals, there is a positive correlation between plasma IL-8 concentrations and glia-related metabolites, such as the total choline in the anterior cingulate cortex and hippocampal myo-inositol, as observed through proton magnetic resonance spectroscopy (1H-MRS) [138]. Moreover, IL-8 might serve as a significant contributor to neuronal loss in Alzheimer’s disease by influencing the release of neurotoxic substances like matrix metalloproteinases (MMPs), and prompting the expression of proteins associated with neuronal cell death, including MMP-2, MMP-9, cyclin D1, and Bim [139].

## 4. IL-8 and Brain Barrier Integrity

The blood–brain barrier (BBB) and the blood–cerebrospinal fluid–brain barrier (BCSFB) collectively act as crucial interfaces between the cerebrovascular system and the brain parenchyma, thereby restoring homeostasis and enhancing the physiological environment of the CNS [140,141].

The BBB plays a vital role in safeguarding the brain and is frequently compromised during various diseases. It primarily consists of brain endothelial cells securely sealed by intercellular junctional structures, including tight junctions [140,141,142,143,144,145]. These endothelial cells, along with other components, such as glia (astrocytes, oligodendrocytes, microglia), neurons, pericytes, and the basement membrane (BM), collectively form the neurovascular unit, ensuring the proper physiological functioning of the CNS [141,146].

The inflammatory response involves complex interactions of various cell types and signaling molecules. Consequently, peripheral inflammation can trigger a neuroinflammatory response involving the BBB, neurons, astrocytes, and microglia. Additionally, the brain itself can release pro-inflammatory mediators upon stimulation [5].

Chemokines act as signaling molecules for immune and nerve cells. They can induce neuroinflammation to protect the organism from pathogens, helping with phagocytosis of debris and apoptotic cells, and contributing to tissue repair. On the other hand, the overexpression of chemokines can disrupt the integrity of the brain barrier and allow immune cells to infiltrate the brain [7,147].

IL-8 has been shown to induce the recruitment of neutrophils to the brain and regulate their adhesion to endothelial cells. This process can result in a significant influx of neutrophils into the subarachnoid space [33]. Once neutrophils breach the BBB, IL-8 induces their degranulation, leading to the release of chemoattractants for T lymphocytes and priming neutrophils for superoxide production, among other potentially neurotoxic molecules [33,148]. Indeed, patients who have had an ischemic stroke have demonstrated an increase in IL-8 plasma levels [149]. In addition, a correlation was observed between the size of the lesion in acute ischemic stroke patients and the levels of IL-8 in the serum [150]. Moreover, the serum level of IL-8 exhibited a positive correlation with the severity of disability in patients who have had an acute ischemic stroke within the initial 48 h post-stroke, as evaluated using the National Institute of Health Stroke Scale (NIHSS) [151].

IL-8 mRNA has been identified within the choroid plexus (CP) [123], an extensively vascularized tissue residing in the brain’s ventricular system [152]. The CP mainly comprises capillary beds, the pia mater, and numerous epithelial cells resting on a basal lamina. Positioned at the interface between the blood and the CSF, the CP plays an essential role in the production of CSF and the formation of the BCSFB [153,154,155,156].

Moreover, the CP is a significant source of biologically active molecules involved in brain development, stem cell proliferation, differentiation, and brain repair [157]. The CP serves as a gateway for the trafficking of immune cells into the CSF and maintains continuous immune surveillance via CD4+ T cells, macrophages, and dendritic cells. It also regulates immune cell trafficking in response to diseases and trauma [153]. Additionally, the CP synthesizes various growth factors, including insulin-like, fibroblast, and platelet-derived growth factors [155].

Inflammation leads to substantial modifications in both the BBB and the BCSFB, causing the disruption of tight junctions (TJs) and the impairment of barrier functionality [158]. TJs are comprised of various proteins, including transmembrane proteins like Occludin, the Claudin family, and the peripheral membrane-associated Zonula occludens (ZOs) family [159,160]. IL-8 has been shown to down-regulate the mRNA expression of Occludin, Claudin-5, and ZO-1. The expression levels of these proteins decrease with higher concentrations and longer durations of IL-8 exposure, displaying a dose- and time-dependent relationship [159].

The CP is implicated in various neurological disorders, including inflammatory, infectious, traumatic, neoplastic, and systemic diseases, as well as autoimmune diseases [161,162]. Additionally, an increased CP volume has been observed in neurodegenerative disorders [163] and psychiatric conditions [164,165].

## 5. IL-8 and CSF

The primary role of CP epithelial cells is to secrete CSF into the brain’s ventricles, and the formation of CSF in the CP depends primarily on the transport of Na^+^, K^+^, Cl^−^, HCO_3_^−^, and H_2_O [162]. The CP mainly contributes to CSF production by allowing free access to the blood compartment through leaky vessels [161]. CSF serves a multitude of functions, including providing mechanical support, acting as a conduit for certain nutrients, eliminating metabolic by-products and waste generated by synaptic activity, and participating in hormonal signaling processes [155,166]. It represents a rich reservoir of various components, including proteins, lipids, hormones, cholesterol, glucose, microRNAs, and numerous other molecules and metabolites, all of which play pivotal roles in modulating a wide spectrum of CNS functions [167].

The identification of the cytokine and chemokine biomarkers within the CSF that correlate with different neuroinflammatory conditions has the potential to serve as a diagnostic tool and offer novel insights into the pathogenesis of these diseases [168,169].

Interestingly, CSF IL-8 levels were found to be elevated in cases of coronavirus disease 2019 (COVID-19) [170,171]. Severe Acute Respiratory Syndrome Coronavirus 2 (SARS-CoV-2) can affect multiple organs, including the brain, leading to neuropsychiatric symptoms and cognitive impairments in patients with COVID-19 [172,173]. It is important to note that SARS-CoV-2 can invade the host’s brain tissue through the olfactory tracts, resulting in anosmia or ageusia [172]. In cases of encephalopathy related to SARS-CoV-2, inflammation within the CNS, induced by IL-8, follows the systemic inflammatory cascade. This inflammation may persist and intensify even after immunotherapy [174]. Moreover, the prolonged production of IL-8 could be associated with extended neurological complications in SARS-CoV-2 infections [174].

Furthermore, elevated CSF levels of IL-8 were observed in individuals with schizophrenia [175], schizophrenia spectrum disorders [176,177], bipolar disorder (BD) [178], major depressive disorder (MDD) [175], in adult patients with autism spectrum disorder (ASD) [179], and patients with Parkinson’s disease and dementia (PDD) [180].

In addition, increased levels of CSF IL-8 were observed in patients with Multiple Sclerosis (MS) [181]. Notably, the time between the first anamnestic episode of focal neurological dysfunction and the diagnosis of relapsing-remitting MS was shown to be a key factor linked to an increase in CSF IL-8 levels. Moreover, a higher risk of disease reactivation, an inadequate response to treatments, and clinical disability were observed in correlation with increased CSF IL-8 levels [182].

Conversely, CSF IL-8 levels were found to be significantly lower in individuals who had attempted suicide [183,184], and were also negatively correlated with symptoms of anxiety in suicide attempters [184].

## 6. CXCL8 Gene and SNPs

The CXCL8 gene is located on chromosome 4q13-21 and comprises four exons and three introns, featuring a unique CAT- and TATA-like structure [113,185,186]. The proximal segment of the CXCL8 promoter includes around 200 nucleotides within the 5′-flanking region of the CXCL8 gene, playing a significant role in regulating the transcription of this gene [187]. Notably, the 5′-flanking region of the IL-8 gene displays distinct sequence dissimilarity compared to other cytokine and acute phase reactant genes [113].

In resting cells, CXCL8 is present in extremely low levels, making it difficult to detect [116,188]. However, the expression of CXCL8 is induced by a range of factors and stressors, with transcription factors NF-κB and activator protein-1 (AP-1) playing key roles in mediating this response. This induction results in a significant increase in CXCL8 expression, typically ranging from 10 to 100-fold [116].

Moreover, genetic polymorphisms, seen in any population, can influence CXCL8 gene expression and may differ between individuals [189,190,191]. For example, the SNP rs4073 (−251A/T), which is located in the promoter region of the CXCL8 gene, influences the transcriptional activity of the CXCL8 gene and has been linked to various diseases [190,191,192]. The A allele of rs4073 is associated with an increased expression of the IL-8 gene and increased IL-8 production. The A/A genotype exhibits the highest values, while the T/T genotype shows the lowest values [191,192,193].

Furthermore, the complex interplay between different SNPs influences CXCL8 gene expression. For instance, in their study, Benakanakere et al., (2016) investigated the effects of stimulating HEK293T cells carrying different genotypes (rs4073 AT, rs2227307 TT, and rs2227306 TC/CC) at the IL-8 locus with the TLR3 agonist poly I:C. They observed that cells with the ATC/TTC haplotype significantly upregulated IL-8 gene expression at both transcriptional and translational levels, leading to enhanced neutrophil transmigration [189].

Table 1 presents four single nucleotide polymorphisms (SNPs) in the Human CXCL8 gene which were reviewed using the SNP database of the National Library of Medicine “https://www.ncbi.nlm.nih.gov/snp/ (accessed on 12 January 2024)”.

CXCL8 gene polymorphisms demonstrate differences in the disease susceptibility between individuals carrying different alleles. For instance, Kang et al., (2015) observed higher frequencies of CXCL8 −251T/A alleles in patients with Alzheimer’s disease (AD), compared to those without AD. However, the significance of these associations was lost after Bonferroni correction [194]. Interestingly, other studies did not find any significant association between patients with AD and the rs4073 polymorphism [195,196,197]. Yet, Infante et al., (2004) discovered a synergistic effect between the rs4073 TT genotype and the IL-1A -889 T allele in patients with AD. Individuals carrying both polymorphisms had about twice the risk of developing AD compared to subjects without these risk genotypes, suggesting a gene–gene interaction [195]. Nonetheless, another study found no interactive effect between the rs4073 and IL-1α -889C/T (rs1800587) polymorphisms in patients with AD [197]. Furthermore, meta-analyses of the rs4073 polymorphism suggested a possible predisposition to AD in individuals of Asian ethnicity [198,199].

In a study by Kamali-Sarvestani et al., (2006), it was noted that there was a significant increase in the presence of the rs4073 TT genotype among patients with Multiple Sclerosis (MS) compared to a group of healthy individuals [200]. Moreover, Dolcetti et al., (2023) discovered a significantly higher concentration of CSF IL-8 in patients with relapsing-remitting MS carrying the rs2227306 T allele and CT/TT genotypes. The study also observed a significant inverse relationship between CSF IL-8 levels and cortical thickness (CT) in individuals carrying the T allele at rs2227306, as assessed through structural MRI measures [201]. In addition, Dolcetti et al., (2023) established a significant positive association between CSF IL-8 levels and patients with a clinical disability in rs2227306 CT/TT assessed with the Expanded Disability Status Scale (EDSS) [201]. Furthermore, the rs2227306 polymorphism was found to be associated with BD I and methamphetamine addiction, but not with BD II, ADHD, MDD, or schizophrenia [202].

Variations in the CXCL8 gene and the resulting changes in IL-8 production could potentially lead to shifts in personality traits that may be associated with an increased risk of suicidal behavior [203]. In their study, Noroozi et al., (2018) determined that the presence of the rs4073 T allele was notably higher in the group of individuals who attempted suicide, in comparison to both the control group and those who completed suicide. Moreover, the haplotype rs4073T/rs2227306C/rs1126647A was significantly less prevalent in the completed suicide group compared to the suicide attempt group. Additionally, the rs4073A/rs2227306T/rs1126647A haplotype was significantly more prevalent in individuals who utilized “hard” suicide methods compared with those who attempted “soft” suicide methods [203].

Janelidze et al., (2015) found that anxiety symptoms were more severe in suicide attempters carrying the rs4073T, and the T allele was more common among females who attempted suicide than in a population-based cohort. Notably, those who attempted suicide and also had rs4073 AA demonstrated a lower median Brief Scale for Anxiety (BSA) score when compared to rs4073 AT and rs4073 TT carriers [184].

Furthermore, Ben Afia et al., (2020) found that the rs1126647 polymorphism within the Tunisian population showed a significant risk for schizophrenia. Notably, females displayed a significant association between the rs1126647 T allele and the T/T genotype, correlating with an elevated risk of paranoid schizophrenia. In males, this predisposition was observed specifically in those carrying the rs1126647 T/T genotype [186]. Moreover, the presence of the rs1126647 T allele and T/T genotype in paranoid schizophrenia was significantly associated with an adult-onset age of 24 years and older. Interestingly, the haplotypes TTT, ACT, and TCT at rs4073/rs2227306/rs1126647, each incorporating the risk allele rs1126647T, were associated with an increased risk for paranoid schizophrenia. However, only the combination of TCT was seen as a risk factor for schizophrenia more generally [186].

In other research, Reyes-Gibby et al., (2013) discovered a notable association between the rs4073 polymorphism and depressed mood, pain, and fatigue in patients with non-small cell lung cancer (NSCLC) in advanced stages (IIIB-IV) of the disease, assessed before cancer treatment. Importantly, individuals carrying rs4073 T/T genotypes were more likely to experience severe depression compared to those with A/T and A/A genotypes. Interestingly, similar rs4073 T/T genotypes among these patients were associated with less severe pain and fatigue compared to carriers of the A/T and A/A genotypes [204].

Additionally, Kim et al., (2013) observed a gene–environment interaction related to the incidence of late-life depression. They identified an association between declining physical health and depression, which was strongest in individuals genetically predisposed to a cytokine-mediated inflammatory response. Notably, they found that rs4073A had a significant modifying effect on the association between physical disorders and the incidence of depression over two years in a Korean population of adults aged 65 and over [205]. However, other studies, including those regarding patients with post-stroke depression [206], depression in breast cancer patients [207], and depression in an elderly Korean population [194], did not find significant associations with the rs4073 polymorphism.

## 7. Maternal IL-8 during Pregnancy and Implications for Offspring

Chemokines can cross the placental and brain barriers, regulating the communication between neurons and microglia in the CNS [208]. During healthy gestation, IL-8 undergoes tight regulation and reaches higher peripheral concentrations during preterm compared to term labor [209]. Additionally, there is a decrease in IL-8 levels from early to later pregnancy, followed by an increase at postpartum [210]. The natural process of childbirth leads to increased circulating IL-8 levels, accompanied by rises in the numbers of neutrophils and monocytes [1]. This elevated post-birth IL-8 level may result from spillage originating in activated vascular endothelial cells or circulating immune cells [1].

Nelson et al., (2006) reported that IL-8 concentrations in newborn infants, both those at preterm and term, surpassed those found in adults [211]. Moreover, Yektaei-Karin et al., (2007) found that the transmigration of IL-8-induced neutrophils in newborns following normal delivery was significantly higher in cord blood compared to neutrophils from Cesarean section births or adult peripheral blood [1].

IL-8 exhibits dual pro-inflammatory and anti-inflammatory roles based on its concentration, suggesting that higher and lower levels of IL-8 might exert opposing effects [212]. This duality implies that IL-8 could play both damaging and defensive roles in the potential demyelination process [212].

Changes in inflammatory cytokines, influenced by environmental factors affecting both maternal and fetal immune systems, can profoundly impact fetal brain development [213]. The exposure of the developing fetus to elevated levels of maternal cytokines has been associated with structural changes in neuroanatomy [214]. Moreover, elevated levels of IL-8 can have significant adverse effects on the developing brain, influencing both its structure and function. Dysregulated IL-8 appears to play a critical role in connecting perinatal systemic inflammation with atypical white matter development in infants born prematurely [215].

Furthermore, increased IL-8 levels in blood samples taken from the umbilical cord or in the infant shortly after birth are strongly associated with visible white matter injury, cerebral palsy, neurodevelopmental challenges, and cognitive impairments in children born prematurely [215]. Remarkably, even among children born at full term, IL-8 stands out as one of the neonatal cytokines most strongly linked to later diagnoses of autism [215]. Interestingly, Jones et al., (2017) found that mothers with higher mid-gestational IL-8 serum levels had a higher risk of having children with autism spectrum disorders with intellectual disabilities. However, mothers of children with developmental delays or autism spectrum disorders without intellectual disabilities had lower mid-gestational IL-8 levels compared to the general population [216].

The increased secretion of IL-8 in Down syndrome may contribute to the observed reduction in postnatal brain growth seen in individuals with this condition. In infants with Down syndrome, IL-8 levels exceed those observed in both subjects with autism and neurotypical control subjects [211]. Additionally, IL-8 appears to amplify the effect of amyloid beta peptide in stimulating the production of IL-6, IL-1β, TNF-α, and COX-2 in cultured human microglia [211,217], suggesting a potential role in the early development of Alzheimer’s neuropathology in Down syndrome [211].

During the second trimester, mothers of offspring with schizophrenia spectrum disorders displayed significantly higher IL-8 levels compared to mothers in the control group [218]. Moreover, within the group of individuals with schizophrenia, the exposure of the fetus to increased maternal IL-8 levels during the second and third trimesters correlated significantly with enlarged ventricular CSF volumes [214]. Additionally, Ellman et al., (2010) reported significant associations between maternal IL-8 levels and reductions in the volumes of the left entorhinal cortex and right posterior cingulate among individuals with schizophrenia. They also observed volumetric reductions that approached significance in the right caudate, bilateral putamen, and the right superior temporal gyrus [214]. Furthermore, Osborne et al., (2022) found notably elevated IL-8 levels, measured in the early third trimester in pregnant women with a previous history of MDD but without depression symptoms during pregnancy, in comparison to pregnant women without an MDD history [219].

Elevated levels of maternal IL-8 in the first trimester were significantly linked to externalizing symptoms (e.g., aggression, impulsivity) and subsequent conduct problems in the offspring [220]. Given that externalizing symptoms in children are tied to cognitive difficulties, it is possible that fetal exposure to IL-8 increases the risk of developing externalizing symptoms through cognitive impairment [220]. Moreover, higher IL-8 levels during early pregnancy (10–18 weeks) were linked to notable decreases in fine motor skills and problem-solving abilities in children at age two [208].

However, prenatal exposure to IL-8 has also been shown to have opposite effects on neurodevelopmental processes. For instance, maternal IL-8 levels during gestation exhibited a positive correlation with verbal abilities while displaying a negative correlation with a child’s spatial abilities [213]. Moreover, reduced IL-8 levels throughout the second and third trimesters were associated with poorer neurocognitive functioning at age seven [221]. Children exposed to lower prenatal IL-8 levels exhibited lower scores in cognitive performance and motor function. Conversely, higher gestational IL-8 levels were associated with improved performance in the Drawing Task and the Tactile Finger Recognition Task [221].

Interestingly, mothers of a higher socioeconomic status (SES) demonstrated higher IL-8 concentrations, while mothers from lower SES families had lower IL-8 levels. Moreover, the decrease in IL-8 levels during pregnancy was associated with compromised child self-regulation [222]. Additionally, increased maternal socioeconomic disadvantage corresponded to noticeably lower IL-8 concentrations during the third trimester, along with a lower ratio of IL-8 to the anti-inflammatory cytokine IL-10. These associations remained unaffected by maternal medical conditions known to disrupt immune responses [223]. Furthermore, lower maternal serum IL-8 concentrations were linked to the presence of neurologic abnormalities in offspring during early life (at ages of 4 months and 1 year) [223].

## 8. IL-8 in Depressive and Bipolar Disorders

Both Bipolar Disorder (BD) and Major Depressive Disorder (MDD) are associated with the activation of the immune-inflammatory response system and the compensatory immune-regulatory system [224,225,226]. BD has been linked to an imbalance in the immune system and low-grade inflammation [227]. This suggests that alterations in IL-8 levels could potentially play a significant role in the pathophysiology of BD.

Tang et al., (2021) established a correlation between increased serum IL-8 levels and impaired functional connectivity (FC) in the right precentral gyrus in unmedicated patients with BD II depression using resting-state functional magnetic resonance imaging (rs-fMRI). This finding suggests that inflammation may contribute to brain functional abnormalities in BD [228]. Moreover, Isgren et al., (2015) found higher CSF IL-8 concentrations in patients with euthymic BD compared to control subjects. Additionally, patients taking lithium or/and antipsychotic medications had even higher IL-8 levels compared to those not taking these medications. Furthermore, IL-8 levels were found to increase with age and with a higher CSF/serum albumin ratio [178]. Notably, in a prospective study, Isgren et al., (2017) did not find an association between CSF IL-8 baseline concentrations and clinical outcomes in patients with BD followed for 6–7 years [229].

The specific pattern of IL-8 alterations appears to be complex. Wang et al., (2016) reported higher IL-8 levels in patients with BD I compared to patients with BD II and other specified BDs with short-duration hypomanic episodes (2–3 days) [230]. In another study, elevated levels of IL-8 in peripheral blood were found only during the depressive phase of BD [231]. Interestingly, in the same study, an association was established between lower blood IL-8 levels and a longer illness duration in BD [231].

Despite the association of both BD and MDD with immune system imbalance, IL-8 levels demonstrate different patterns. For instance, serum IL-8 levels were exclusively elevated in BD patients, but not in MDD patients [232]. Additionally, plasma IL-8 levels were directly associated with bipolar depression when compared to MDD [224]. This suggests that IL-8 may serve as a potential biomarker to differentiate between MDD and BD, especially in cases of atypical clinical presentations [232]. Moreover, IL-8 testing may even hold prognostic value in identifying resilience or a risk of depression [233].

Despite the above-described evidence, inconsistencies regarding IL-8 levels in BD exist. For instance, Barbosa et al., (2013) reported decreased plasma levels of IL-8 in patients with BD I compared to controls [234]. Conversely, some studies found no significant differences in IL-8 levels between patients with BD patients and controls [235,236].

Research indicates that immune dysregulation plays a significant role in depression [237], and numerous studies have evaluated the correlation between IL-8 levels and disease progression as well as treatment response. For instance, higher baseline plasma levels of IL-8 in breast cancer survivors were linked to a reduced risk of incident and recurring major depression [233]. Moreover, elevated IL-8 plasma levels demonstrated an association with a lower severity of depressive symptoms in depressed patients, while treatment-induced increases in IL-8 predicted a positive treatment response [238]. Conversely, a decline in serum IL-8 levels was associated with depression [239]. Notably, patients with MDD who positively responded to antidepressant treatment exhibited lower baseline IL-8 levels than those who did not respond [240].

In a study by Zou et al., (2018), patients with MDD showed significantly lower serum levels of IL-8 compared to controls, revealing linear correlations between IL-8 and the severity of depression [241]. They also observed significant linear correlations between IL-8 levels and anxiety levels in patients with comorbid anxiety disorders. Thus, higher IL-8 levels were associated with lower scores on the Hamilton Depression Rating Scale (HAM-D) and the Hamilton Anxiety Rating Scale (HAM-A) [241].

Furthermore, a higher baseline level of IL-8 in plasma was correlated with less pronounced increases in depressed mood and feelings of social disconnection [238]. Interestingly, Cai et al., (2023) suggested that elevated serum IL-8 levels might correspond to improvements in delayed memory and visuospatial/constructional function in patients with MDD [242]. However, it is important to note that this positive association was not universally observed. Baune et al., (2008) found that higher IL-8 levels in healthy elderly individuals were associated with poorer performance on specific neuropsychological tests related to memory, processing speed, and motor function. Notably, IL-8 levels in these individuals were not associated with general cognitive function as assessed by the Mini-Mental State Examination (MMSE) [243].

Intriguingly, the concentrations of IL-8 appear to be influenced by several factors, including sex differences, which were reported in various studies exploring IL-8 findings. For example, an elevated plasma concentration of IL-8 was inversely related to the HAM-D score in females, but this relationship was not detected in males [244]. Additionally, Moriarity et al., (2019) found a correlation between higher initial plasma IL-8 levels and a reduction in depressive symptoms at a follow-up 31 months later, specifically in adolescent males [245].

Moreover, treatment response may also exhibit sex-specificity. For instance, baseline plasma levels of IL-8 and changes in IL-8 related to treatment with electroconvulsive therapy (ECT) were associated with improvements in depression in females, but not males [246]. Similarly, lower baseline plasma levels of IL-8 and subsequent increases in IL-8 were specifically correlated with improved depression in females treated with ketamine [247]. These findings highlight the need for personalized treatment approaches based on individual IL-8 profiles and possible gender-specific responses.

Interestingly, physical activity has also been shown to influence IL-8 levels. For instance, in patients with MDD, serum concentrations of IL-8 significantly increased following vigorous exercise, while no changes occurred after light and moderate exercise. Importantly, depression severity did not seem to impact the acute inflammatory response to exercise [248].

It is noteworthy that research on IL-8 remains multifaceted and ongoing, and there are conflicting findings regarding IL-8 levels in depression. Several studies have reported significantly higher serum IL-8 levels in patients with MDD compared to controls [212,249,250]. Another study found no association between plasma IL-8 levels and depressive symptom severity in physically active individuals after SARS-CoV-2 infection and a follow-up after 3 months [251]. In contrast, Ogłodek (2022) reported an increase in IL-8 serum concentration with depression severity. Notably, the highest increase in IL-8 levels was seen in a group of patients with severe depression that co-occurred with Post-traumatic Stress Disorders (PTSDs) [252]. Additionally, Suneson et al., (2023) found significantly higher plasma IL-8 levels in patients with difficult-to-treat depression compared to controls [253]. Importantly, Szałach et al., (2022) identified that serum IL-8 values exceeding 19.55 pg/mL were associated with a 10.26 likelihood ratio of developing treatment-resistant depression [254].

## 9. IL-8 and BDNF

Brain-derived neurotrophic factor (BDNF), a member of the neurotrophin family, plays a significant role in neuronal survival and differentiation during development and maintains high expression levels in the adult brain [255,256]. BDNF participates in many functions, including neuronal migration, synaptic structure regulation, and neurotransmitter release, all of which are vital for brain circuits regulating memory, learning, emotions, sleep, and appetite [257]. Moreover, BDNF regulates synaptic transmission and activity-dependent plasticity and is predominantly found in various regions of the brain, including the hippocampus, amygdala, cerebellum, and cerebral cortex, with hippocampal neurons exhibiting the highest BDNF levels [256]. Therefore, given the wide array of functions attributed to BDNF, it is not surprising that it is associated with a multitude of neuropsychiatric conditions [258,259,260].

In neonates without brain injury born between 23- and 41-weeks of gestation, there is a significant gestation-dependent increase in serum BDNF levels (3.7%) and IL-8 levels (8.9%) for every week of gestation [261]. However, BDNF concentrations do not show any association with the first 7 days of life (DOL), while IL-8 levels increase with each DOL by 18.9% [261]. Additionally, BDNF concentrations exhibit a remarkable increase with age, with a progressive and significant ascent from an average of 740 pg/mL in very preterm control infants to nearly 5500 pg/mL in adults [211].

Interestingly, BDNF can directly contribute to anti-inflammatory effects on microglia by regulating cytokine responses, which in turn can impact neurons. BDNF’s priming of microglia may reprogram the inflammatory state, leading to alterations in neuron–microglia interactions [262]. It is worth noting that both human fetal and adult microglial cells produce IL-8 in response to lipopolysaccharide (LPS), along with two key cytokines involved in initiating an inflammatory response, namely IL-1β and TNF-α [33].

Newborns diagnosed with neonatal encephalopathy exhibited increased plasma levels of IL-8 and decreased BDNF levels, in comparison to healthy newborns. Notably, there was an inverse correlation between BDNF levels and the encephalopathy grade, while IL-8 levels were inversely linked to motor outcomes [263]. Additionally, research has found that patients with schizophrenia have significantly lower BDNF levels and higher IL-8 serum levels compared to individuals without schizophrenia [264,265]. Interestingly, a positive correlation was observed between BDNF and IL-8 levels [264,265]. This could suggest that a relative increase in BDNF levels possibly acts as a compensatory response in patients with schizophrenia, although it might not be sufficient to counteract the inflammatory damage caused by increased cytokines like IL-8 [264,265]. Notably, Xiu et al., (2019) discovered a negative correlation between reduced serum BDNF levels and executive function in patients with chronic schizophrenia. They observed an interaction between low BDNF and high IL-8 concentrations, which were positively correlated with executive dysfunction as measured by verbal fluency tests (VFT) and Wisconsin card sorting tests (WCST) in patients with schizophrenia [265].

Moreover, Wang et al., (2016) noted elevated IL-8 plasma levels in individuals with BD, while BDNF levels did not exhibit significant differences compared to a control group [230]. In contrast, another study found no significant difference in IL-8 plasma levels between subthreshold bipolar disorder (SBD) and BD-II at baseline and following a 12-week mood stabilization treatment. However, in the SBD group, the study revealed markedly lower baseline BDNF plasma levels which remained low even after 12 weeks of treatment, despite similar treatment responses between the two groups [266].

Furthermore, Liou et al., (2023) reported a significant difference in IL-8 and BDNF plasma levels between patients with BD and those in a control group. Moreover, patients with BD and comorbid alcohol use disorder (AUD) displayed higher IL-8 levels compared to patients with BD without AUD [267]. Additionally, lower BDNF levels were associated with decreased performance on cognitive assessments, while plasma IL-8 levels in patients with BD demonstrated a significant negative correlation with the number of completed categories in the Wisconsin Card Sorting Test (WCST) [267].

## 10. IL-8, ROS, and Oxidative Stress

ROS act as signal transduction molecules activating IL-8 and significantly regulating the production of this cytokine [58,268]. While ROS play a crucial role as cellular signaling molecules for normal biological processes, their excessive production can induce harm to various cellular components and functions, potentially disrupting physiological equilibrium [269,270,271].

The etiology of various diseases has been linked to an imbalance between ROS production and the protective antioxidant defenses of cells, particularly in conditions involving inflammation or ischemia-reperfusion, where excessive ROS generation occurs [121,269,272]. In the brain, ROS can trigger cellular damage contributing to cognitive dysfunction and the development of neuropsychiatric conditions [273,274,275].

Oxidative stress occurs when the production of ROS and other oxidants surpasses the body’s antioxidant defenses and ability to neutralize them [276,277]. This is particularly concerning for brain neurons, which rely heavily on oxidative phosphorylation for energy and are vulnerable to oxidative stress [278]. Indeed, even minor disturbances in the redox equilibrium during neural development, when combined with genetic or environmental susceptibilities, can significantly impact neurogenesis, neuronal differentiation, and neural connectivity [276]. ROS generate free radical oxidation products that can interact with cellular metabolites, potentially causing cell death through apoptosis or necrosis [279]. One significant consequence of ROS overproduction and oxidative damage is DNA alteration, potentially leading to permanent mutations and other genomic instabilities [278]. It is important to note that ROS are generated by various sources and are mostly produced in mitochondria as byproducts of cellular metabolism [280,281].

The role of IL-8, ROS, and oxidative stress becomes even more critical when considering the impact of viral infections such as COVID-19. The inflammatory response triggered by SARS-CoV-2 leads to the release of cytokines, chemokines, and ROS, causing the disruption of TJs and compromising the brain barrier’s integrity [282]. Furthermore, coronavirus infection disrupts mitochondrial regulation, leading to a reduction in Adenosine Triphosphate (ATP) synthesis and the activation of NADPH oxidase, thereby contributing to ROS production [283]. Moreover, COVID-19 affects the morphological features and distribution of astrocyte and microglia cells [284]. Notably, the SARS-CoV-2 receptor, angiotensin-converting enzyme 2 (ACE2), is expressed by astrocytes and microglia [285].

Interestingly, Clough et al., (2021) observed an increase in IL-8 gene expression in microglial cells treated with both the SARS-CoV-2 spike protein and heat-inactivated SARS-CoV-2, compared to untreated controls. Moreover, higher IL-8 cytokine expression was seen in microglia treated with heat-inactivated SARS-CoV-2 compared to those treated with the SARS-CoV-2 spike protein [286]. Activated microglia and astrocytes can release ROS and cytokines, contributing to sustained neuroinflammatory responses and neuronal damage [282,287,288].

Furthermore, when stimulated by IL-8, neutrophils migrate to the inflammation site, degranulate and release lysosomal enzymes that not only damage pathogens but also inadvertently harm nearby tissue by oxidizing cellular components like lipid membranes, proteins, and DNA [289]. Furthermore, activated neutrophils contribute to oxidative stress through the production of ROS via NADPH oxidase during the respiratory burst [290]. Prolonged oxidative stress can facilitate the development and persistence of inflammation by activating transcription factors that modify the expression of various genes and proteins, including pro-inflammatory cytokines [291]. Consequently, in severe cases, excessive ROS production by deregulated neutrophils can escalate the local inflammatory response to a systemic level [292].

The oxidative stress and cytokine storm create a vicious cycle, escalating inflammation and ultimately contributing to multi-organ failure in severe COVID-19 cases [286]. Neurons, being particularly vulnerable to such inflammatory and oxidative damage, are increasingly implicated in the development of Long COVID, a post-infection condition characterized by lingering neurological and psychiatric symptoms [283]. Patients with Long COVID often report fatigue, cognitive impairments, sensory dysfunctions, headaches, post-exertional malaise, and experience mood disorders. These symptoms can persist for months or even years, affecting memory, language, processing speed, and executive function [283,293,294,295,296,297,298,299].

Importantly, individuals with pre-existing health comorbidities or neuropsychiatric vulnerabilities are at an increased risk of developing severe COVID-19 and long-term post-COVID neurological and psychiatric consequences [300]. This could be partly explained by the oxidative stress and elevated levels of IL-8 which are found in patients with neuropsychiatric conditions. For example, Wu et al., (2021) observed decreased activities of superoxide dismutase (SOD) and glutathione peroxidase (GPx), along with elevated levels of malondialdehyde (MDA) and IL-8 in individuals with chronic schizophrenia. Moreover, the combined effects of IL-8 with MDA or SOD were found to correlate with executive function in patients with chronic schizophrenia [301]. Similarly, Wei et al., (2020) discovered a connection between oxidative stress parameters and serum BDNF levels in individuals with chronic schizophrenia, revealing lower GPx and SOD activities, reduced BDNF levels, and elevated MDA levels compared to those of controls [276]. Enzymatic antioxidants such as SOD, catalase (CAT), GPx, and non-enzymatic antioxidants like glutathione (GSH) serve as a defense mechanism, reducing ROS activity and maintaining cellular redox balance [281,302,303]. Additionally, MDA, a lipid peroxidation by-product, is commonly used as a marker of oxidative stress [304,305,306].

It was shown that the production and concentration of IL-8 can be suppressed by antioxidants, notably N-acetylcysteine (NAC) [31,57,58,108,109,110,307,308]. NAC possesses both anti-inflammatory and antioxidant properties, facilitating the replenishment of glutathione and the scavenging of free radicals [309,310,311,312]. Glutathione is essential for intracellular and intercellular signaling in the brain, and its depletion contributes to oxidative stress, leading to neuronal metabolic disturbances and consequential alterations in synaptic signaling [313,314,315,316,317,318,319,320]. Adjunctive therapy with NAC has demonstrated positive outcomes in various neuropsychiatric conditions [321,322,323,324,325,326,327,328,329,330].

## 11. Discussion

Many factors, including genetic predisposition, environmental conditions, and immune responses, contribute to neuropsychiatric conditions. The host’s immune system significantly modulates both physiological and pathological processes, and inflammatory immune responses have been linked to disease severity and treatment efficacy [331,332]. Peripheral immune and inflammatory cells can migrate to the brain through the compromised BBB, and proliferate at sites of inflammation, directly exacerbating the inflammatory response or amplifying it via glial and neuronal activation [333].

Chemokines play a crucial role in the interaction between the immune and nervous systems, being involved in various brain-related processes such as neurodevelopment, neurogenesis, neuromodulation, synaptic transmission, neuroendocrine homeostasis, brain barrier integrity, neuroinflammation, and stress response [19,20,334,335,336].

IL-8 belongs to the CXC chemokine subfamily and is produced early in the inflammatory response. It can remain active for a prolonged period, making it particularly relevant to the long-term inflammatory alterations observed in neuropsychiatric conditions [337,338,339]. IL-8 is secreted by various cell types and is released when exposed to an inflammatory trigger. Furthermore, numerous cell types possess receptors for IL-8, and upon binding, they generate molecules with both local and systemic activity [35,340].

The variety of cellular origins for IL-8 highlights the pleiotropic nature of its functions. While IL-8 is essential for the host’s defense mechanism due to its influencing neutrophil activation and trafficking, its prolonged presence in the bloodstream during inflammatory conditions can result in varying degrees of tissue damage, and contribute to disease-related processes, such as fibrosis, angiogenesis, and tumorigenesis [9,340].

Considering the various roles of IL-8, it is significant that psychological stress can cause an increase in IL-8 secretion, which could potentially worsen disease processes [341]. Experiencing chronic stress during early life can lead to long-lasting immune, endocrine, neural, and inflammatory alterations, contributing to a broad range of neuropsychiatric conditions later in life [342].

The production of IL-8 has been linked to depression, negative affect traits, and perceived stress [289]. Interestingly, individuals with strong social support and larger social networks have lower IL-8 plasma levels, particularly among healthy midlife adults [289]. Additionally, a positive association has been found between IL-8 levels and fewer years of education, indicating a potential link with lower socioeconomic statuses [289]. Suarez et al., (2004) found that both the severity of depressive symptoms, measured by the 21-item Beck Depression Inventory (BDI), and hostility, assessed using the 50-item Cook–Medley Hostility (Ho) scale, independently and synergistically increase IL-8 expression in healthy premenopausal women [343].

Stressful work conditions elevate urinary IL-8, which indicates its potential use as a reliable biomarker of stress [344,345]. For example, Dutheil et al., (2013) reported that emergency physicians on 24 h shifts showed a nearly doubled level of urinary IL-8 compared to controls or those on 14 h shifts. This prolonged immune response persisted for at least three days after the 24 h shifts, despite a day of rest following the 24 h shifts. Notably, older age, stressful work conditions, and long shifts were associated with increased IL-8 levels [344]. Additionally, IL-8 levels correlated positively with exposure to life-and-death emergencies and were exacerbated by sleep deprivation and poor sleep quality during shifts [344]. Furthermore, Fukuda et al., (2008) observed that hospital acute care department nurses with higher professional stress had higher urinary IL-8 levels compared to chronic care departmental nurses [345].

Prolonged and intense exposure to stress can disrupt the body’s energy balance and reduce adaptation mechanisms, resulting in adverse effects on overall well-being [346]. Psychological stress is associated with an elevated production of oxidants, exacerbating oxidative stress within the body [347]. This oxidative burden is particularly pronounced in individuals experiencing chronic stress, as the continuous activation of the HPA axis perpetuates oxidative damage over time [348]. Moreover, Kim et al., (2021) reported that acute psychosocial stress can induce a noticeable elevation in oxidative stress within less than 2 h [349]. Such oxidative stress arises from an imbalance between ROS production and antioxidant defenses and is often exacerbated by various cellular stressors that elevate ROS levels or reduce the body’s ability to neutralize them [350,351].

Given the brain’s high metabolic activity and oxygen consumption, it is especially vulnerable to oxidative stress, which can significantly impair CNS functions and contribute to neuroinflammation and neurodegeneration [352,353,354]. Moreover, the interplay between oxidative stress and inflammatory responses adds complexity to stress-induced pathophysiological alterations. Indeed, oxidative stress and neuroinflammation have the potential to mutually reinforce each other [355]. ROS accumulation within cells can disrupt intracellular signaling, leading to the dysregulation of inflammatory processes [353]. In addition, elevated ROS levels upregulate the production of IL-8 by promoting NF-κB signaling [356]. Thus, ROS and oxidative stress contribute to IL-8 production [60,357,358]. IL-8 exhibits remarkable stability and prolonged biological activity, resisting proteolysis and denaturation, while its mRNA maintains sustained expression in the presence of stimulating agents, underscoring its significant biological impact [290]. Excessive IL-8 correlates with heightened NF-κB translocation and reduced glutathione levels [359]. Additionally, IL-8 facilitates the ROS metabolism and induces ROS production [35,360]. Therefore a potential positive feedback loop may exist between oxidative stress and IL-8, fueling each other and contributing further to cellular damage.

IL-8 plays a crucial role in attracting neutrophils, and a deficiency in CXCL8 can impair the movement of these cells to tissues [361]. For example, children suffering from microcephaly because of Congenital Zika Syndrome showed a significant decrease in serum IL-8 levels, leading to an impairment in leukocyte migration [361]. On the other hand, a rise in the absolute neutrophil count and IL-8 levels in the bloodstream were linked to the severity of COVID-19, wherein plasma IL-8 levels were associated with mortality [362]. Critically ill patients with COVID-19 demonstrated a high neutrophil-to-lymphocyte ratio associated with elevated levels of ROS, which could lead to tissue damage, thrombosis, and the dysfunction of red blood cells, thereby contributing to the severity of COVID-19 [292].

The nervous and immune systems engage in complex communication, which is regulated by multiple mechanisms. This interaction can be disrupted by various triggers such as infections, autoimmune diseases, peripheral and systemic inflammation, traumatic brain injuries, environmental toxins, and stress, potentially leading to neuroinflammation [363,364,365,366,367,368,369,370,371,372,373,374,375].

One important diagnostic tool for measuring this inflammatory response is IL-8. However, the use of this marker needs to be personalized, as diverse populations exhibit variances not only in their susceptibility to and progression of diseases, but also in the levels of inflammatory markers, such as IL-8. For instance, Mayr et al., (2007) reported that healthy young volunteers of African descent had higher average IL-8 levels compared to their Caucasian counterparts [376]. Moreover, plasma IL-8 levels demonstrated an inverse correlation with neutrophil counts in individuals of African descent and in combined groups, but not in those who were Caucasian alone [376]. Additionally, Mayr et al., (2007) reported a lower oxidative burst capacity in stimulated neutrophils among volunteers of African descent [368].

It was also observed among African American women that both stress/distress and poor sleep quality had notable impacts on the production of proinflammatory cytokines during the postpartum period [377]. Specifically, in African American women, but not in White women, evaluations conducted at 7–10 weeks postpartum revealed that poorer sleep quality, heightened parenting stress, increased depressive symptoms, and elevated general perceived stress were all associated with greater LPS-stimulated IL-8 production [377].

The observed variations in IL-8 levels across individuals and different populations can be partially attributed to genetic polymorphisms. The CXCL8 gene, which encodes IL-8, exhibits several functional polymorphisms that may influence IL-8 production [191,378]. For example, rs4073 (−251A/T) has been linked to inflammatory diseases, and the rs4073 A allele has an association with increased IL-8 production [193,379,380]. Additionally, the A/A genotype may reduce the threshold for IL-8 synthesis [380].

Wacharasint et al., (2012) reported that critically ill Caucasian patients with the rs4073 A/A genotype had an increased risk of a PaO(2)/FiO(2) < 200 (the PaO2/FiO2 ratio is the ratio of arterial oxygen partial pressure), and demonstrated greater IL-8 mRNA expression than those with the A/T or T/T genotypes [190]. Additionally, Hildebrand et al., (2007) suggested that rs4073 polymorphism can influence the severity of the inflammatory response following multiple traumas. They found that the rs4073 A/A genotype showed a significantly longer duration of mechanical ventilation after trauma compared to genotype T/T [379]. In contrast, the rs4073 T allele, which is linked to lower IL-8 production, was associated with the severity of microcephaly in children with congenital Zika syndrome [381]. In addition, Zhao et al., (2020) identified the rs4073 T/T genotype as a potential risk factor for sepsis in full-term neonates [382].

Ethnic groups exhibit varying distributions of genetic polymorphisms. For instance, Fujihara et al., (2007) reported that Ovambos and Gambians displayed the lowest rs4073 T allele frequencies at 8% and 10%, respectively, while those who are Japanese had the highest T allele frequency at approximately 80%, with the T/T genotype being 67% [191].

Interestingly, the same genetic polymorphisms can have varying impacts on disease vulnerability across populations. For example, Wang et al., (2013) discovered a significant association between the A/A and A/T genotypes of the rs4073 polymorphism and increased oral cancer risk among Caucasian populations, while no statistically significant association was found among Asian populations [383]. In contrast, Zhang et al., (2019) found a significant association between the rs4073 A allele increasing coronary artery disease (CAD) risk in a Chinese population, but this association was not observed in Caucasians [384]. Zhang et al., (2021) conducted a meta-analysis revealing that CXCL8 rs4073 polymorphisms may affect a predisposition to Alzheimer’s disease in people who are Asian, but not in people who are Caucasian [198]. This suggests that CXCL8 gene polymorphism can have a distinct influence not only on various disease states but also on health outcomes across diverse populations. Such variability indicates the presence of additional factors that may modulate these effects. Indeed, environmental factors can interact with the genome by modifying epigenetic mechanisms that regulate gene expression [385].

Furthermore, it is important to note that there can be an interplay between SNPs of the CXCL8 gene, which may result in a combined effect when inherited together. For instance, the haplotype of CXCL8 rs4073T/rs2227306C/rs1126647T is associated with an increased risk for schizophrenia [186]. Additionally, a significant effect can be seen in gene–gene interactions. For example, Ghazy (2023) discovered a significant correlation between the simultaneous presence of IL-8 rs2227306C and IL-6 rs1800795G alleles in an individual and an increased risk of severe COVID-19 outcomes. Conversely, individuals carrying the IL-8 rs2227306T and IL-6 rs1800795C alleles were found to have a reduced risk of severe COVID-19 [386]. In summary, considering individual and ethnic differences is crucial when interpreting inflammatory markers for an accurate diagnosis and personalized treatment plans.

## 12. Conclusions

IL-8 emerges as an important mediator in the crosstalk between the body’s defense mechanisms and the nervous system, demonstrating a profound influence on a multitude of psychoneuroimmunological processes. This underscores its potential role in the pathogenesis of various neuropsychiatric conditions. The current data highlight the complex interplay between IL-8, ROS, environmental factors, psychosocial stressors, and genetic backgrounds, underscoring the need for further studies to fully understand these potential influences and their implications for personalized medicine. Polymorphisms in the CXCL8 gene can impact IL-8 production, potentially leading to diverse research findings related to disease susceptibility, progression, and severity across different populations. Therefore, further research on IL-8 is essential to enhance our knowledge of its diverse roles in physiologic and pathologic processes, and to consider individual variations. Its potential use as a biomarker could ultimately lead to more personalized and effective strategies for diagnosing and treating patients with neuropsychiatric conditions.

## Figures and Tables

**Table 1:** CXCL8 gene SNPs.

SNP	Alleles	Gene: Consequence	Genomic Position
rs4073	A > C/A > G/A > T	CXCL8: 2KB Upstream Variant	chr4:73740307 (GRCh38.p14)
rs1126647	A > C/A > T	CXCL8: 3 Prime UTR Variant	chr4:73743328 (GRCh38.p14)
rs2227306	C > T	CXCL8: Intron Variant	chr4:73741338 (GRCh38.p14)
rs2227307	T > C/T > G	CXCL8: Intron Variant	chr4:73740952 (GRCh38.p14)

## Data Availability

Data sharing is not applicable to this article. No new data were created or analyzed in this study.
